# Anterior Cruciate Ligament Reconstruction in a Rabbit Model Using Silk-Collagen Scaffold and Comparison with Autograft

**DOI:** 10.1371/journal.pone.0125900

**Published:** 2015-05-04

**Authors:** Fanggang Bi, Zhongli Shi, An Liu, Peng Guo, Shigui Yan

**Affiliations:** Department of Orthopedic Surgery, the Second Affiliated Hospital, School of Medicine, Zhejiang University, Hangzhou, China; Texas A&M University Baylor College of Dentistry, UNITED STATES

## Abstract

The objective of the present study was to perform an in vivo assessment of a novel silk-collagen scaffold for anterior cruciate ligament (ACL) reconstruction. First, a silk-collagen scaffold was fabricated by combining sericin-extracted knitted silk fibroin mesh and type I collagen to mimic the components of the ligament. Scaffolds were electron-beam sterilized and rolled up to replace the ACL in 20 rabbits in the scaffold group, and autologous semitendinosus tendons were used to reconstruct the ACL in the autograft control group. At 4 and 16 weeks after surgery, grafts were retrieved and analyzed for neoligament regeneration and tendon-bone healing. To evaluate neoligament regeneration, H&E and immunohistochemical staining was performed, and to assess tendon-bone healing, micro-CT, biomechanical test, H&E and Russell-Movat pentachrome staining were performed. Cell infiltration increased over time in the scaffold group, and abundant fibroblast-like cells were found in the core of the scaffold graft at 16 weeks postoperatively. Tenascin-C was strongly positive in newly regenerated tissue at 4 and 16 weeks postoperatively in the scaffold group, similar to observations in the autograft group. Compared with the autograft group, tendon-bone healing was better in the scaffold group with trabecular bone growth into the scaffold. The results indicate that the silk-collagen scaffold has considerable potential for clinical application.

## Introduction

The anterior cruciate ligament (ACL) plays a major role in the stability and normal kinematics of the knee joint [1]. Rupturing or tearing of the ACL can cause instability of the knee joint, leading to dislocation, injury to other ligaments, or osteoarthritis [2, 3]. Due to its limited capacity for regeneration, the ACL heals poorly when the ruptured ends are sutured back together [4]. It is estimated that more than 100,000 cases of ACL reconstruction are performed in the United States each year [5]. Grafts are needed for ACL reconstruction in clinical settings. Current graft types include autografts, allografts, and synthetic grafts. ACL reconstruction with autografts is the current gold standard, but it has considerable drawbacks, such as prolonged surgery, long rehabilitation time, donor site complications, and decreased range-of-motion (ROM) [4, 6]. Allografts also have disadvantages, such as a higher failure rate compared with autografts, immunogenic responses, and risk of disease transmission [7]. Permanent synthetic grafts can involve complications, including long-term rupture, foreign-body response, and poor tissue integration [8]. Recently, ligament tissue engineering has emerged as a promising means of overcoming the shortcomings of autografts, allografts, and synthetic grafts [9].

The ideal tissue engineering graft for ACL reconstruction should provide immediate joint stability, and it should also gradually degrade and diminish in strength as the ligament regenerates and remodels [10–12]. The scaffold is a key component of tissue engineering, as it can affect cellular behavior and the formation of the extracellular matrix (ECM). Thus, the selection of suitable material and the manner of reconstruction are important [8, 11]. In recent years, collagen, silk, composite materials, and biodegradable polymers have been studied as potential scaffolds for ACL reconstruction. Collagen is the main component of ECM. It drew early interest because of its biocompatibility, biodegradability, and ability to induce cell differentiation. However, it cannot provide sufficient mechanical support, and it diminishes rapidly over time, even after cross-linking treatments [8]. Although both cross-linking of collagen with chemical reagents or ultraviolet, and braid-twist scaffold designs were shown to improve the mechanical properties of collagen scaffold, the mechanical strength was still less than desired [13, 14]. Polymeric scaffolds such as polyglycolic acid (FGA), polydioxanone (PDS), polylactic-co-glycolic acid (PLGA), and poly L-lactic acid (PLLA), have shown excellent mechanical strength [12, 15–17], but their lack of signaling molecules and hydrophobicity reduce cell adhesion, proliferation, and subsequent function [18]. To overcome the weaknesses inherent in some biomaterials, several modification methods have been developed, and various composite materials have been created. Hansson *et al*. coated chitosan with arginine-glycine-aspartic acid (RGD) peptide to increase cell adhesion [19]. Other researchers developed alginate-chitosan composite scaffolds to promote fibroblast growth and type Ⅰcollagen production in vitro and in vivo [20–23]. Silk has received research attention as a promising scaffold for tissue engineering of ligaments owing to its desired biocompatibility, strength, toughness, and elasticity [24, 25]. Altman and Kaplan were the first to use native silk fibroin fibers as 3D scaffolds to reconstruct an ACL, and the twisted scaffold provided sufficient mechanical support for the human knee joint [26]. However, ingrowth of the newly regenerated connective tissues was inhibited by the limited internal space in the braided or twisted fiber scaffold [9, 27], restricting neoligament regeneration. Although these biomaterials and their composites have shown interesting possibilities, further research is needed to identify their potential for ligament tissue engineering.

Here, a new scaffold was designed using a synergistic combination of knitted structure silk fibroin and collagen matrix; the intention was that the silk fibroin would provide mechanical strength, and the collagen matrix would occupy the internal space provided by the knitted scaffold for cell ingrowth. This study was designed to test the hypothesis that "a silk-collagen scaffold would increase the rate of ligament regeneration compared with an autograft". This hypothesis was examined by (1) preparing a combined scaffold that incorporated knitted silk fibroin mesh and collagen matrix, and (2) examining neoligament regeneration and tendon-bone healing through radiographic, biomechanical, and histological methods. The purpose of the present study was to determine whether the silk-collagen scaffold is suitable for ACL reconstruction.

## Materials and Methods

### Scaffold fabrication

**Fig 1:**
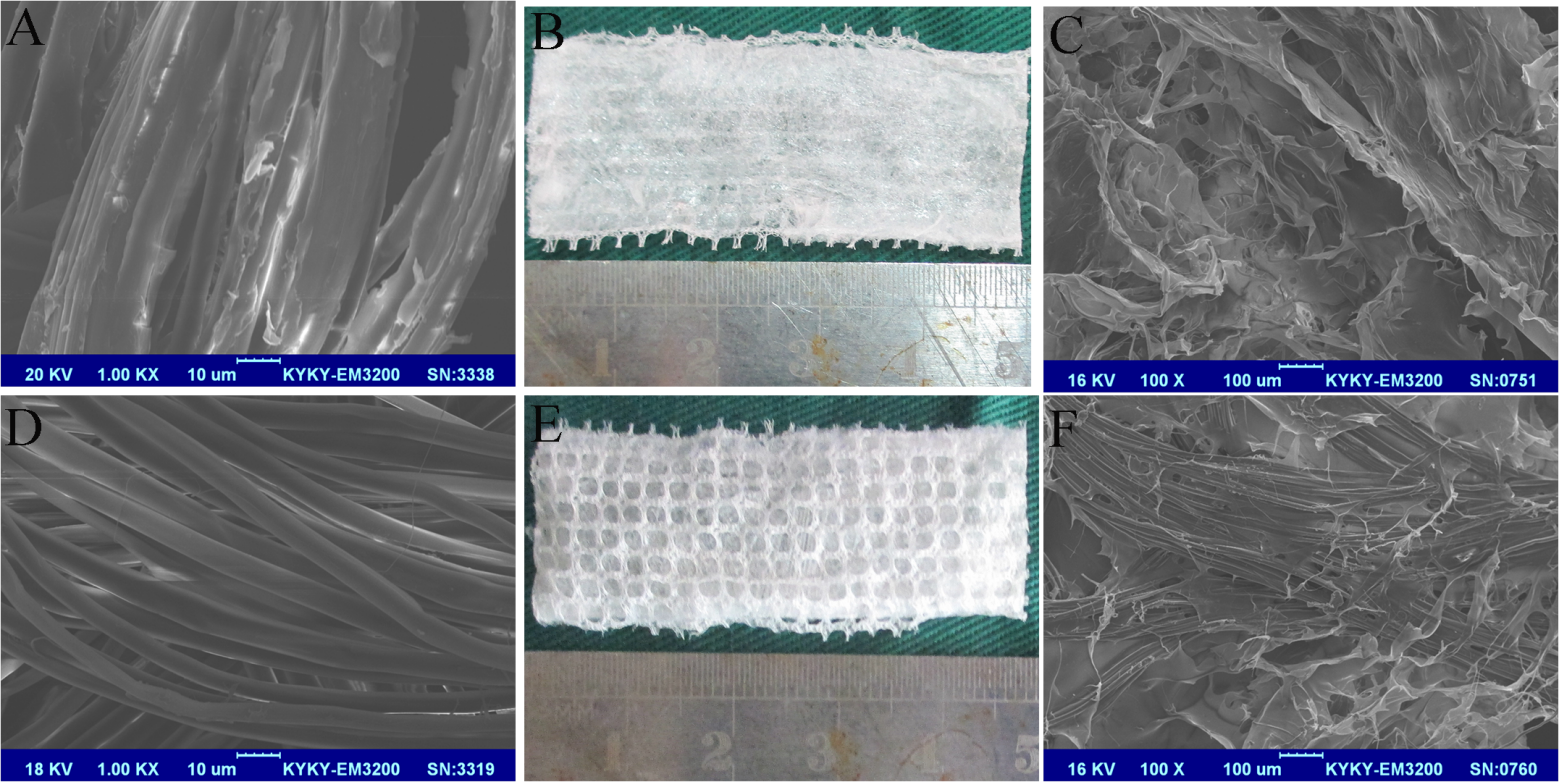
The structure of raw silk fibers and silk fibroin under SEM (×1000): sericin, the glue-like protein coating the fibroin is shown (A); after complete degumming, the underlying silk fibroins with a smooth surface and an average diameter of about 10 μm are shown (D). Gross observation of the silk-collagen scaffold: (B) the collagen sheet adheres to (E) the knitted silk fibroin mesh with pores about 1.0 mm in diameter. Structure of the silk-collagen scaffold under SEM (×100): (C) collagen sheet side and (F) the silk fibroin mesh side.

Zhejiang Cathaya International Co. Ltd. provided the raw *Bombyx mori* silk fibers. Twelve yarns (one filament/yarn) of silk fiber were used to fabricate the silk mesh on a knitting machine. Plain knitted silk scaffolds were manufactured with 21 stitches per centimeter; the pore size was approximately 1×1 mm [28]. Next, sericin, the glue-like protein coating silk fibroin (Fig 1A), was extracted using 0.02 M Na_2_CO_3_ aqueous solution at 90°C and 100°C for 60 min, with the process repeated three times [26]. After degumming was completed, the underlying smooth-surfaced silk fibroins with an average diameter of about 10 μm were observed under the scanning electron microscope (SEM, Fig 1D).

Neutral salt and dilute acid extractions were used to isolate and purify the collagen matrix from the Achilles tendons of pigs [29]. The sericin-extracted knitted silk fibroin mesh (2×5 cm), which was kept in its original flat condition, was immersed in an acidic collagen solution (type I, PH3.2, w/v 1%, 1 mm depth), and then frozen at -80°C for 12 h. It was then subjected to freeze-dry processing under vacuum (Heto PowerDry LL1500) for 24 h, which allowed the formation of collagen microsponges [30]. Then, the scaffold was dehydrated in a vacuum oven at 105°C and 30 mTorr for 24 h for dehydrothermal (DHT) cross-linking [31]. Finally, the scaffold was sterilized by irradiation with cobalt-60. All subsequent analyses were carried out to evaluate the hybrid scaffolds.

### Experimental design

Forty male New Zealand white rabbits (2.5–3.0 kg, 12 weeks old) were used in the experiment. The study protocol was approved by the University of Zhejiang Institutional Animal Care and Use Committee. Rabbits were randomly divided into two main groups (scaffold group and autograft group), and the left hind leg was used to reconstruct the ACL. The semitendinosus tendon and silk-collagen scaffold were used to reconstruct the ACL in the autograft group and scaffold group, respectively. Ten rabbits in each group were sacrificed at 4 weeks, and the same number at 16 weeks postoperatively. Half of the specimens in each group (n = 5) were used to evaluate tendon-bone healing by micro-CT and biomechanical test. The remaining specimens (n = 5) were used to evaluate neoligament regeneration using hematoxylin and eosin (H&E) staining and immunohistochemical staining, as well as tendon-bone healing by H&E and Russel-Movat pentachrome staining.

### Surgical protocol

**Fig 2:**
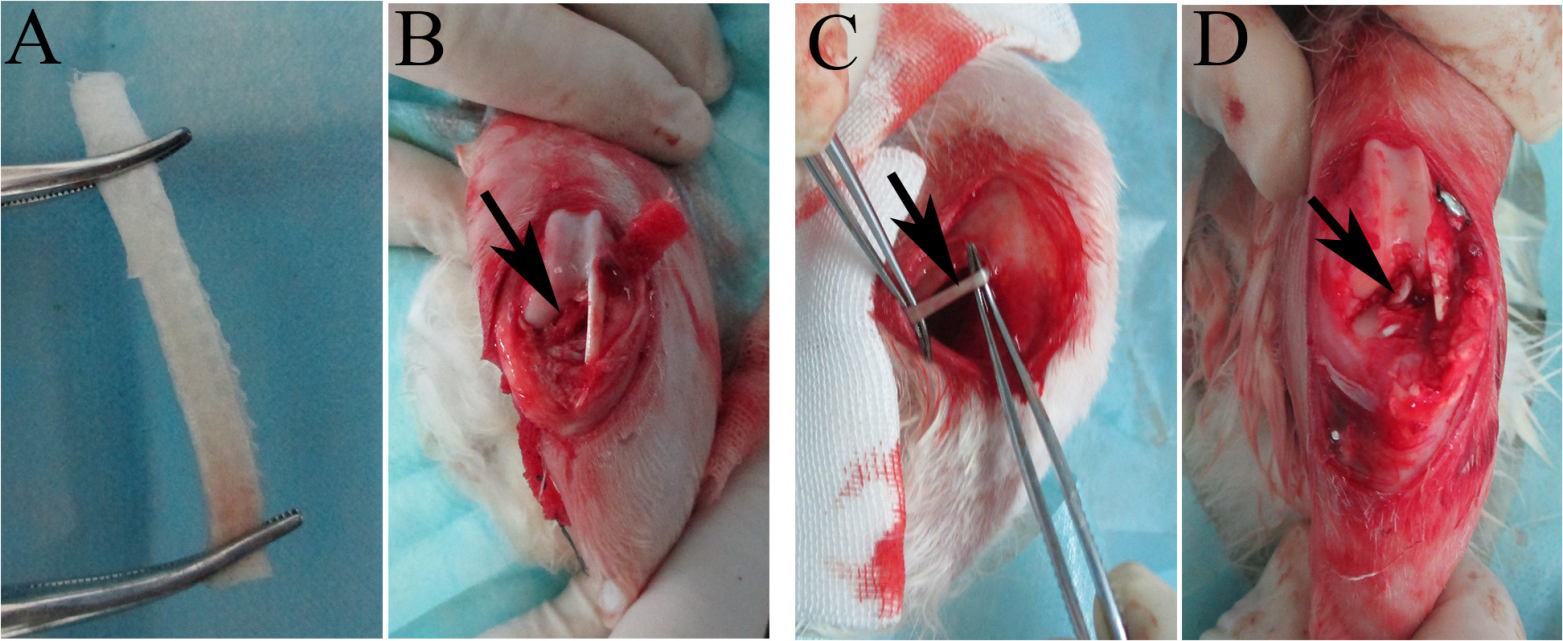
A rolled-up shaft of silk-collagen scaffold, (A), with the two ends clamped by forceps. (B) Gross observation of ACL reconstruction (the black arrow points to the silk-collagen graft). (C) The auto-semitendinosus tendon (arrow) was harvested before exposing the knee joint. (D) Gross observation of ACL reconstruction (the black arrow points to the autograft).

All surgeries were performed by one of the authors (Bi). After achieving general anesthesia with an intravenous injection of 3% pentobarbital sodium solution (30 mg/kg), the left hind leg of the rabbit was shaved, disinfected, and draped. A longitudinal incision about 3 cm in length was made at the medial border of the patellar tendon to expose the knee joint. For the rabbits in the autograft group, the semitendinosus tendon was harvested before exposing the knee joint (Fig 2C). In the scaffold group, the silk-collagen scaffold was carefully rolled up along the short axis to make a tightly wound shaft 2.0 mm in diameter and 50 mm in length (Fig 2A). The native ACL was excised, and tibial and femoral tunnels from the ACL attachment site were created with a 2.0 mm drill. The graft was then introduced into the bone tunnels, and both ends were fixed using sutures tied to screws in the tibia and femur. The knee joint was flexed at 30° with the graft in slight tension when the procedure was done (Fig 2B and 2D). The wound was sutured in layers. All rabbits were allowed to move around in their cages without restriction after the operation.

### Evaluation of the neoligament regeneration

Paraformaldehyde (4%; Sangon, Shanghai, CHN) in 0.1 M phosphate-buffered saline (PBS) was used to fix the specimens (n = 5 from each group at each time point) for 24 h immediately after their collection. The graft in the knee joint cavity was dissected (the remaining bone samples were used to evaluate tendon-bone healing) and then processed by dehydration and embedded in paraffin. The slices were sectioned at 5μm along the longitudinal axis of the graft, and stained with H&E for conventional light microscopy. To analyze ligament regeneration of the autograft and the silk-collagen scaffold, monoclonal antibody for tenascin-C (Abcam, UK) was used for immunohistochemistry. Briefly, slices were deparaffinized with xylene and a graded alcohol solution according to standard procedures. After antigen retrieval and blocking following the protocol of the Antigen Retrieval and Sealing Solution kit (Boster, CHN), primary monoclonal antibody from mice was diluted in 5% bovine serum albumin (BSA) solution at the recommended dilution rate (1:1000). It was then incubated with the section overnight at 4°C. Slices were incubated with a horse radish peroxidase (HRP)-labeled goat anti-mouse secondary antibody (Dawen, CHN) diluted 1:1000 in 5% BSA solution for 2 h at room temperature, and then washed with PBS. The regenerated ligament was evaluated based on the presence of tenascin-C.

### Evaluation of tendon-bone healing

#### Histology

Ethylenediaminetetraacetic acid (10%; EDTA) in PBS was used for decalcification of the bone samples until the bone could be sectioned easily with a blade. After dehydration and embedding, the samples were sectioned along the longitudinal axis of the bone tunnels. H&E staining and Russell-Movat pentachrome staining were performed to evaluate osteogenesis at the tendon-bone interface using light microscopy.

#### Micro-CT scan

The femur-graft-tibia complex (n = 5 from each group at each time point) were stored at -80°C immediately after collection for subsequent micro-CT scanning. Before testing, the specimens were thawed overnight at 4°C. The areas of the vertical plane across the axis of the bone tunnel were measured at a depth of 5 mm from the tibial joint surface (micro-CT, 36 μm thickness; Skyscan1176, Bruker, Belgium). The X-ray levels were 65 kV and 370 μA. Each area was measured three times with image analysis software (ImageJ; National Institutes of Health), and the average value was used for analysis.

#### Biomechanical test

**Fig 3:**
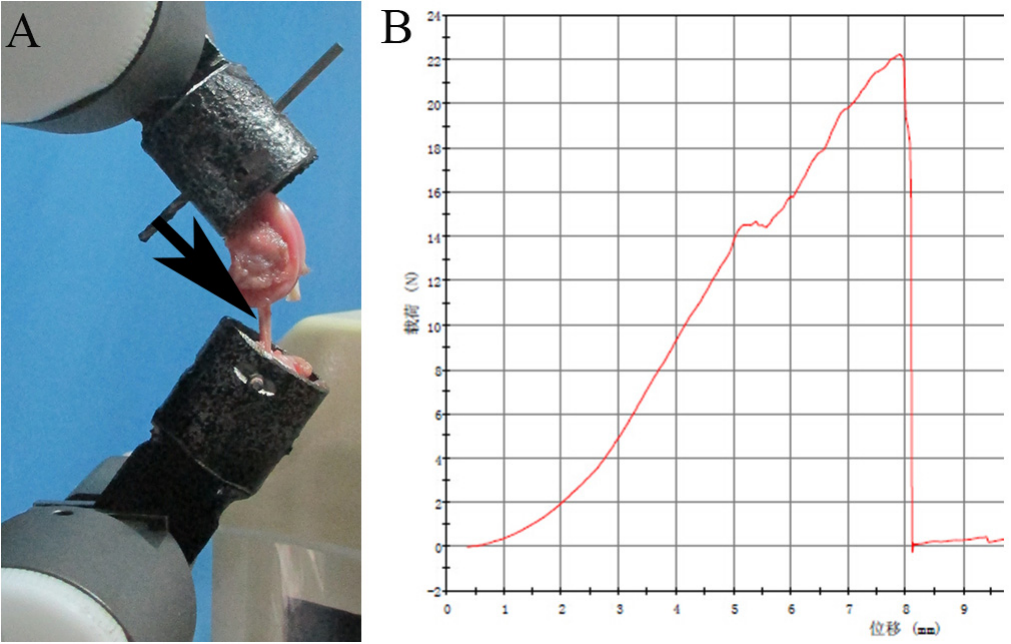
The femur and tibia were fixed in custom iron tubes, (A), and clamped in an Instron machine to perform the biomechanical test (the black arrow points to the graft). (B) A typical load-deformation curve obtained by the biomechanical test.

The five femur-graft-tibia complex samples for each group were used for biomechanical test immediately after the micro-CT scan. All ligaments except the graft and soft tissue were dissected and removed. The femur and tibia were fixed with custom iron tubes and clamped in an Instron 553A material testing system (Instron, USA, Fig 3A) at 45° flexion. The biomechanical test was carried out by continuously increasing the tensile displacement at a speed of 20 mm/min. The specimens were kept moist with normal saline (NS) solution during the test. The representative load-deformation curve comprised the toe region, linear region, microfailure region, and failure region. The failure load could be directly obtained from the curve, and the slope of the linear region was considered to indicate stiffness (Fig 3B).

### Statistical analysis

All statistical analyses were carried out using SPSS 16.0 software, and p<0.05 was considered to indicate statistical significance. The data in the present study including ROM, bone tunnel area and biomechanical properties, are expressed as mean ± standard deviation (SD). Independent-sample t-tests were used to detect differences.

## Results

### Characterization of the silk-collagen scaffold

The silk-collagen scaffold had two distinctive sides: a silk fibroin side and a collagen sheet that adhered to the knitted silk fibroin mesh. The pores of the knitted silk fibroin mesh were about 1.0 mm in diameter. The collagen sponge was distributed over the surface, and it penetrated into the loops of the knitted silk fibroin mesh. The penetration of collagen into the scaffold resulted in a fuzzier surface (Fig 1B and 1E). SEM revealed that the collagen sponge formed in the interspaces of the knitted silk fibroin mesh, with the collagen sponge and silk fibroin alternately chained (Fig 1C and 1F).

### Gross observation

**Fig 4:**
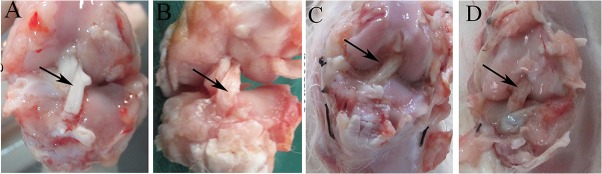
Macroscopic view of grafts in the autograft group (A, B) and scaffold group (C, D) at 4 weeks (A, C) and 16 weeks (B, D) postoperatively. The arrows point to the grafts in the knee cavity.

The performance of the experimental rabbits was evaluated through observations of locomotion, ROM, and the drawer test at 2 and 4 weeks after surgery. The knee ROM of the scaffold group was significantly greater than that of the autograft group at 2 weeks postoperatively (*P*<0.05). However, there was no significant difference in the flexion angle ROM between groups at 4 weeks postoperatively (*P*>0.05, Table 1). No rabbit had a positive drawer test. When gathering the specimens, no gaps were seen between grafts and bone tunnels in each group. The sites of attachment to the bones were all intact macroscopically. Gross examination revealed no obvious fibrous tissue growth into grafts in the autograft group at 4 and 16 weeks postoperatively (Fig 4A and 4B). However, at 4 weeks postoperatively, we observed that fibrous tissue had infiltrated into the regenerated ligament in the scaffold group, and the loose silk fibers were held tightly in a bundle by the abundant ingrowth tissue. Highly vascularized connective tissue filled the space between the graft and the bone tunnel. At 16 weeks postoperatively, more newly formed fibrous tissue was observed, which fully occupied the space among silk fibers and encapsulated the scaffold (Fig 4C and 4D).

**Table 1:** Range of motion (left hind leg) at 2 and 4 weeks postoperatively in the autograft and scaffold groups.

Time point	Scaffold group			Autograft group			*P* value
	Minimum	Maximum	Mean±SD	Minimum	Maximum	Mean±SD	
2 weeks	85	140	112.3±16.6	50	135	96.5±20.7*	0.01
4 weeks	105	140	136.3±8.1	95	140	136.3±5.1	1.00

* Significant difference between groups.

### Evaluation of the neoligament regeneration

**Fig 5:**
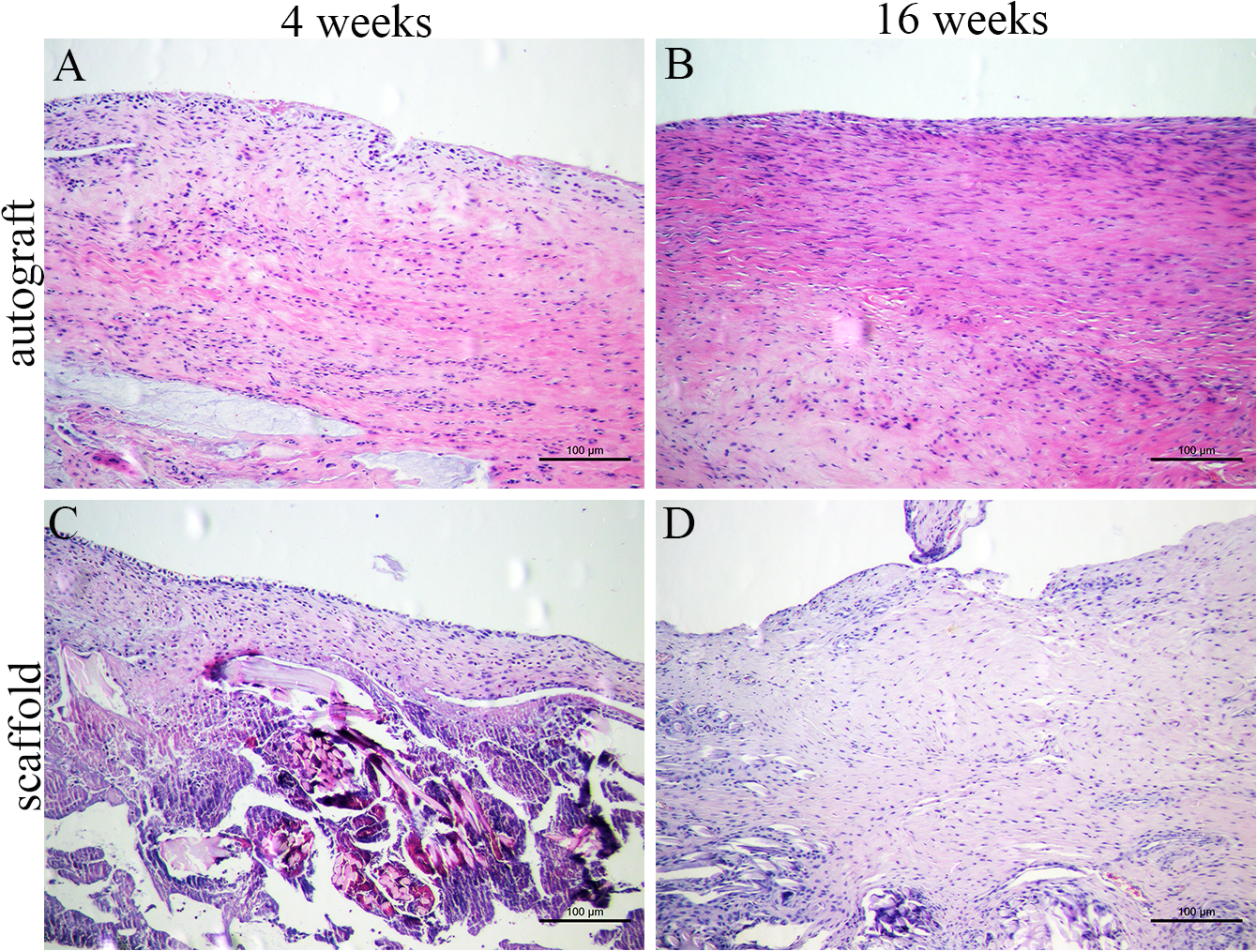
Histological observation of grafts in the autograft group (A, B) and scaffold group (C, D) by H&E staining at 4 weeks (A, C) and 16 weeks (B, D) postoperatively.

**Fig 6:**
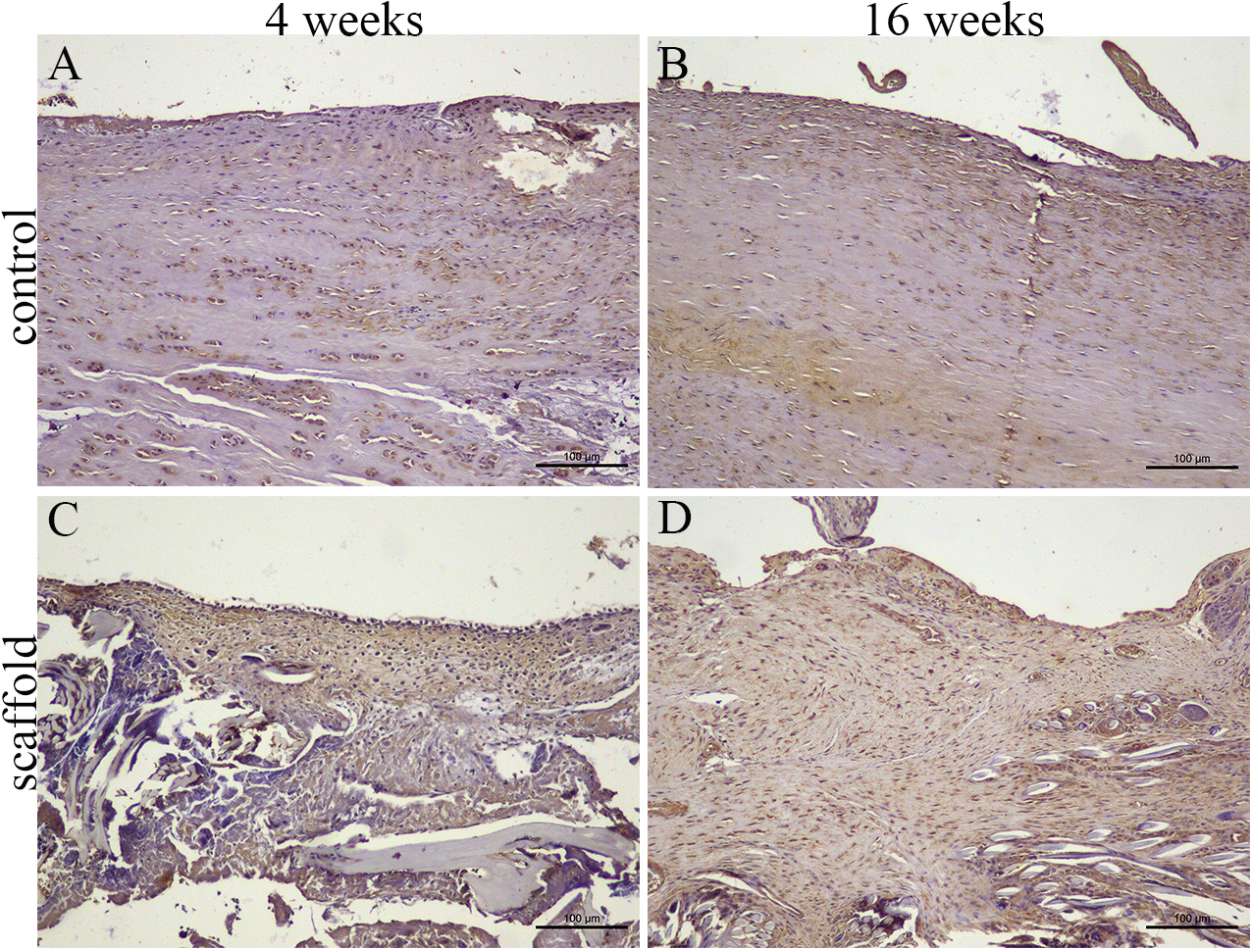
Histological observation of grafts in the autograft group (A, B) and scaffold group (C, D) by immunohistochemistry staining specific for tenascin-C to evaluate ligament regeneration at 4 weeks (A, C) and 16 weeks (B, D) postoperatively.

H&E staining and immunohistochemical staining were performed to evaluate the cell infiltration and tenascin-C production of the intra-articular grafts in each group. In the autograft group, the graft showed considerable cell invasion (Fig 5A and 5C). In the scaffold group, the fibroblast-like cells were mostly distributed in the surface layer at 4 weeks postoperatively, and few cells were seen in the inner part of the graft. At 16 weeks postoperatively, host cells had invaded the core part of the scaffold. Fibroblast-like cells appeared denser and more regular in the scaffold group than in the autograft group (Fig 5B and 5D). Immunohistochemical staining for tenascin-C was strongly positive in the newly regenerated tissue at 4 and 16 weeks postoperatively in the scaffold group; this was similar in the autograft group (Fig 6A, 6B, 6C and 6D).

### Evaluation of tendon-bone healing

#### Histology

**Fig 7:**
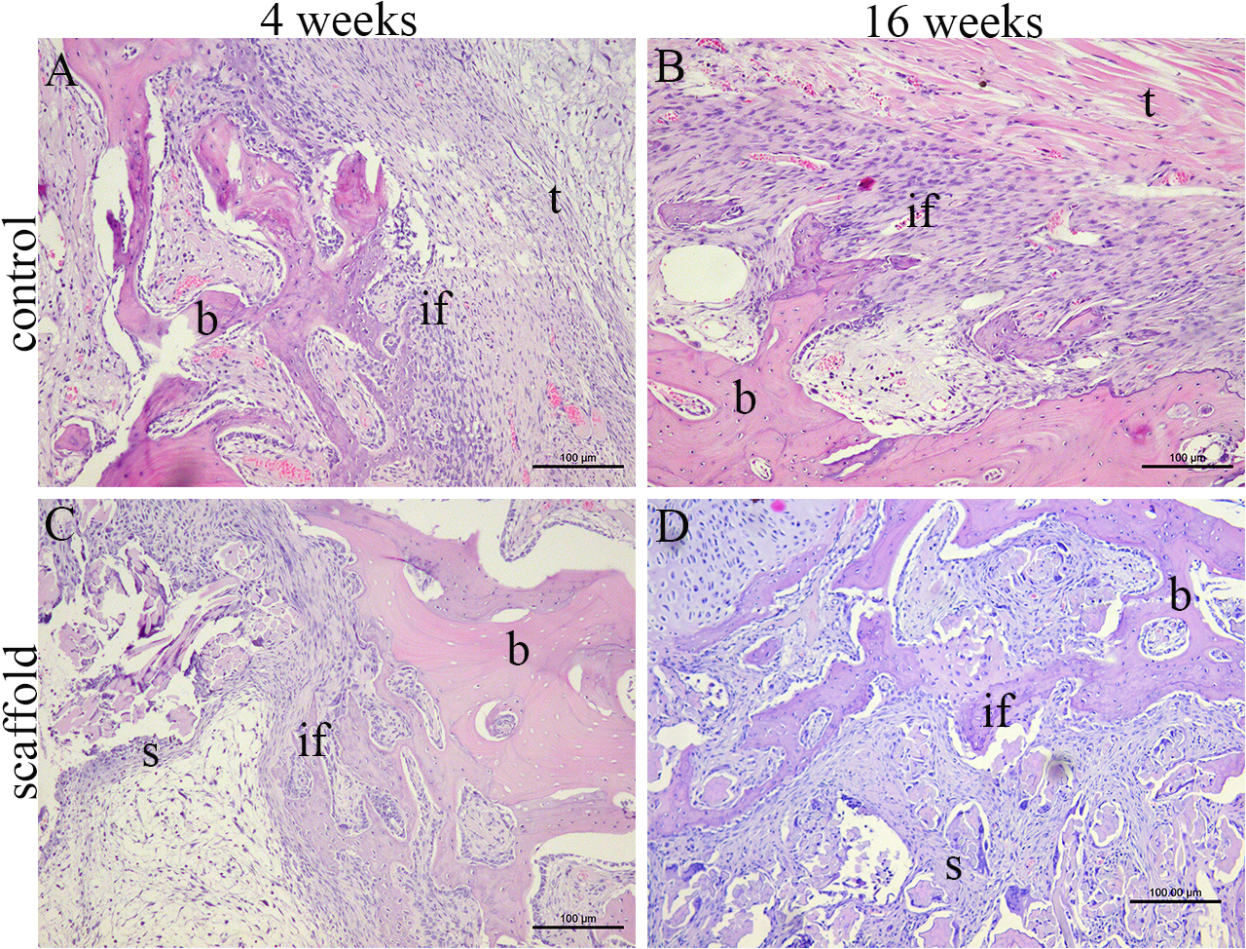
Histological observation of tendon-bone healing in the autograft group (A, B) and scaffold group (C, D) by H&E staining at 4 weeks (A, C) and 16 weeks (B, D) postoperatively. (b: bone, if: interface, t: autograft tendon, s: silk-collagen scaffold.)

**Fig 8:**
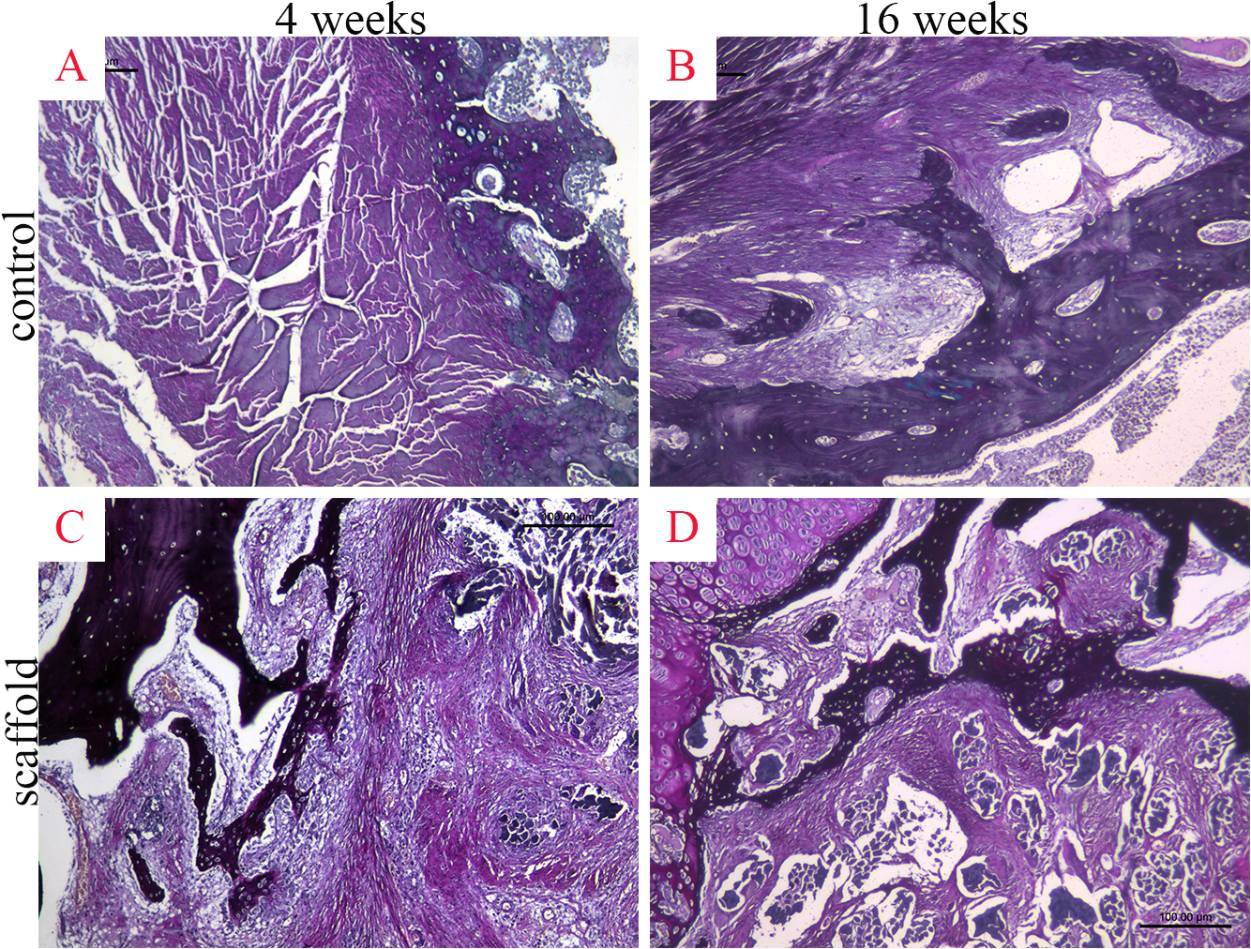
Histological observation of tendon-bone healing in the autograft group (A, B) and scaffold group (C, D) by Russell-Movat pentachrome staining at 4 weeks (A, C) and 16 weeks (B, D) postoperatively.

At 4 weeks postoperatively, organized connective tissue and a thin layer of chondrocytes were observed at the tendon-bone interface, but no obvious bone formation was observed in either group (Figs 7, 8A and 8C). By 16 weeks postoperatively, trabecular bone had grown into the inside of the graft in the scaffold group, and in the autograft group, typical Sharpey’s fibers had formed at the tendon-bone interface (Figs 7, 8B and 8D).

#### Micro-CT scan

**Fig 9:**
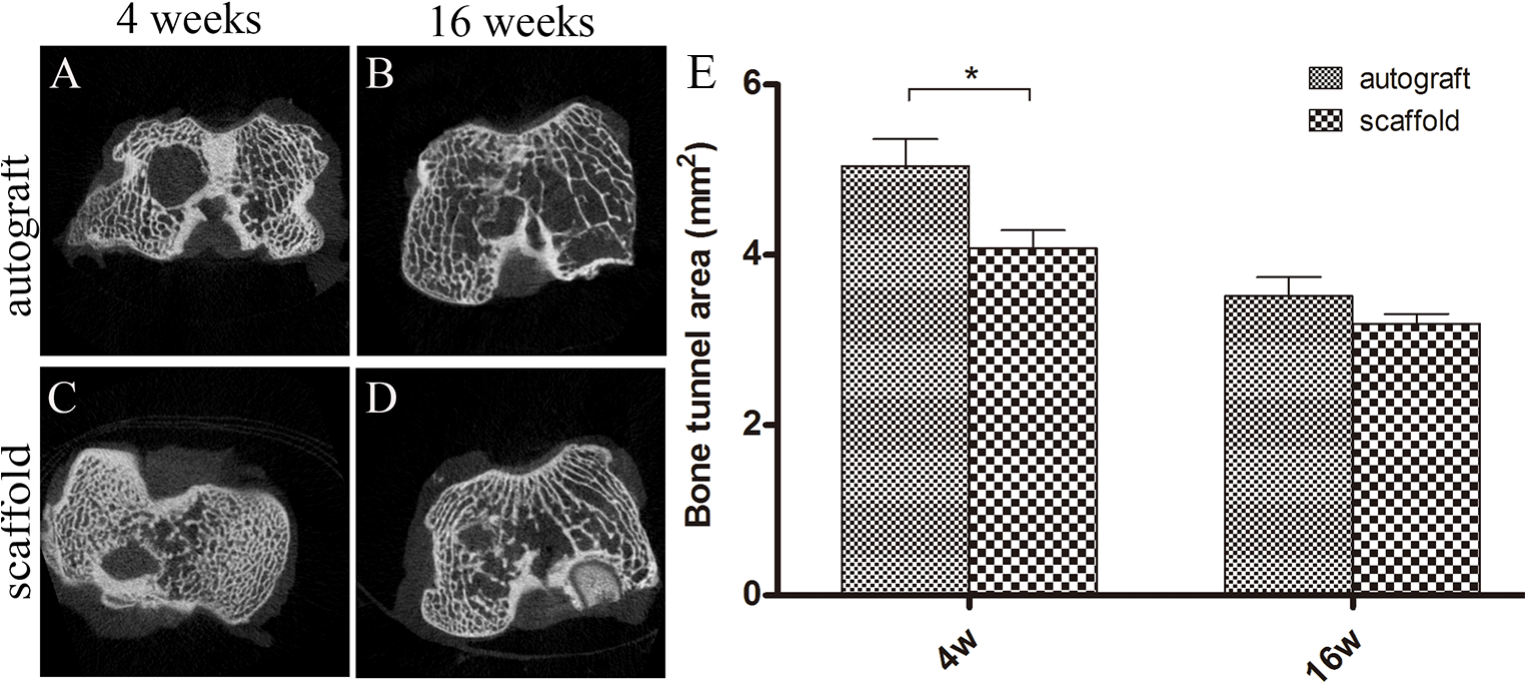
Micro-CT scan images of the autograft group (A, B) and scaffold group (C, D) at 4 weeks (A, C) and 16 weeks (B, D) postoperatively. (E) The average bone tunnel area in the scaffold group was significantly smaller than that in the autograft group at week 4. There was no significant difference between the two groups at week 16. * Significant difference between groups.

The cross sectional images vertical to the longitudinal axis of bone tunnel were reconstructed with micro-CT at high resolution. The newly formed mineralized tissue of the bone tunnel could be detected by screening all slices from each sample. In both groups, there was no obvious indication that mineralized tissue had formed within the bone tunnels at 4 weeks postoperatively (Fig 9A and 9C); however, at 16 weeks postoperatively, obvious signals suggesting newly formed mineralized tissue were detected in the bone tunnels of both groups (Fig 9B and 9D). The average bone tunnel area in the scaffold group was significantly smaller than that in the autograft group at 4 weeks postoperatively (autograft, 5.05±0.70 vs. scaffold, 4.08±0.47 mm^2^; *P<*0.05, n = 5). There was no significant difference between the two groups 16 weeks postoperatively (autograft, 3.52±0.50 vs. scaffold, 3.19±0.25 mm^2^; *P>*0.05, n = 5; Fig 9E).

#### Biomechanical test

**Fig 10:**
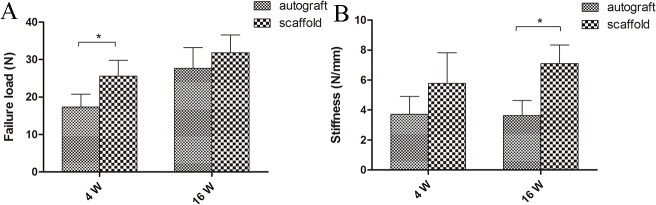
Statistical evaluation of differences in failure load (A) and stiffness (B) between the autograft group and scaffold group at 4 and 16 weeks postoperatively. * Significant difference between groups.

The biomechanical test was successfully performed on specimens in each group. The failure load and displacement were read from the load-deformation curve. With extension of the grafts, the curves changed smoothly from a flat to a steep slope. The failure load in the scaffold group was significantly higher than that in the autograft group at 4 weeks postoperatively (autograft, 17.33±3.43 vs. scaffold, 25.63±4.17 N; *P*<0.05, n = 5). At week 16, there was no significant difference in the failure load between the two groups (autograft, 27.64±5.56 vs. scaffold, 31.85±4.74 N, *P*>0.05, n = 5; Fig 10A). There was no significant difference in stiffness between the two groups at 4 weeks postoperatively (autograft, 3.72±1.19 N/mm vs. scaffold, 5.78±2.04 N/mm; *P*>0.05, n = 5). At week 16, the stiffness in scaffold group was significantly greater than that of the autograft group (autograft, 3.63±1.01 N/mm vs. scaffold, 7.09±1.25 N/mm; *P*<0.05, n = 5, Fig 10B).

## Discussion

Researches on the use of grafts for ACL reconstruction have focused on tissue engineering strategies to promote ligament regeneration and tendon-bone healing [32]. The current study demonstrated that a silk-collagen scaffold could regenerate the ACL after implantation. The regenerated ligament was infiltrated by a large number of fibroblast-like cells, which were similar to those found in the autograft group. The tensile strength met the mechanical needs of daily activities, and the tendon-bone interface showed good healing at 16 weeks postoperatively. These data suggest that the silk-collagen scaffold is a suitable biomaterial for ACL reconstruction.

The ECM plays an important role in providing mechanical support, guiding tissue ingrowth, and maintaining homeostasis during the ligament regeneration process. An ideal tissue engineering scaffold for ligament regeneration should mimic not only the geometric structures of ECM, but also its biological functions [33]. In recent years, silk has emerged as a potential material for ligament tissue engineering because of its unique mechanical properties and inherent suitability for weaving, braiding, and knitting. Altman *et al*. developed a silk scaffold with a hierarchical structure for a ligament reconstruction [26, 34]. Such scaffolds have mechanical properties similar to those of the native ACL, and they demonstrate viscoelastic properties that play important roles in the prevention of damage due to fatigue and creep. Chen and colleagues found that modifying silk fibroin with short polypeptide chains; e.g.,arginine-glycine-aspartic (RGD), could increase cellular attachment and proliferation [35]. Murphy *et al*. modified the functional groups on the tyrosine residues in the silk fibroin by a diazonium coupling reaction and found that more hydrophilic silk derivatives promoted cell proliferation [36]. However, the neoligament tissue regeneration was restricted by the limited internal space in twisted or braided fiber scaffolds. In the present study, the incorporated collagen microsponges increased surface area, which facilitated cell adhesion, proliferation, and ECM production. Collagen accounts for 60–85% of tendon dry weight and plays an important role in tensile resistance [37]. Collagen gel and sponge have often been used in research on tendon and ligament tissue engineering because of their excellent biocompatibility [38–40]. However, their poor mechanical properties and rapid degradation preclude their use as a graft intended to provide mechanical strength for ligament tissue engineering [41–43]. Although knitted collagen scaffolds provide sufficient mechanical strength and internal connective space, the internal space decreases when the scaffolds undergo heavy physical loading.

Therefore, to take advantage of the excellent biocompatibility of collagen matrix while preserving the internal space of the knitted silk fibroin scaffold, these two biomaterials were combined by pouring a collagen solution on silk fibroin sheets. The space between the silk fibroin was dominated by collagen, which provided a point of attachment for cells rather than allowing them to leak out from the scaffold. The histology demonstrated that the collagen sponge in the internal space promoted cell infiltration and connective tissue regeneration in the scaffold. Four weeks after implantation, fibroblast-like cells were distributed on the surface of the graft. At 16 weeks postoperatively, fibroblast-like cells had already invaded the core of the scaffold.

In the present study, the scaffold was found to provide sufficient mechanical strength to withstand the daily activities of the experimental rabbits. At 2 and 4 weeks after surgery, no rabbit in the scaffold group had a positive drawer test. The silk fibroin fibers were the main components transferring the load to the scaffold, and functional tissue engineering emphasized the interactions between cells and the ECM. The silk scaffold with a hierarchical structure developed by Altman had similar mechanical properties to the native ACL, with a maximum load of 2337±72 N, elastic modulus of 354±26 N/mm, and strain at failure of 38.6±2.4%. Mechanical properties are an important consideration in ligament regeneration tissue engineering. The grafts should maintain a certain tensile strength while the new tendon is forming. In our study, the tensile strength of the silk-collagen scaffold could be increased by adding silk fibers to the scaffold as needed. A previous study demonstrated that dynamic mechanical stimulation was beneficial to tissue-engineered tendons [44]. Mechanical stimulation can influence cell activity and induce cell differentiation into a functional tissue assembly, and the regenerated tissue can meet necessary mechanical requirements. Mesenchymal stem cells (MSCs) can attach, proliferate, and differentiate on silk scaffolds [26]. Some progenitor/stem cells, including MSCs and synovial stem cells, have recently received attention due to their potential for promoting osteogenesis and angiogenesis and thereby improving the tendon-bone healing process [45–47]. Fan *et al*. demonstrated that stem cells produced more collagen-I, collagen-III, and tenascin-C when co-cultured with silk scaffolds for 7 or 14 days [48]. In our study, the tenascin-C, which is an extracellular matrix glycoprotein of sections of intra-articular grafts, was evaluated, and we found that immunohistochemical staining for tenascin-C in the scaffold group was similar to that in the autograft group. Tenascin-C has a highly restricted gene expression pattern, and is prominently expressed in actively remodeling tissue [49]. H&E staining revealed that fibroblast-like cells in the scaffold group were similar to those in the autograft group at the same postoperative time point. The results suggested that grafts in the scaffold group displayed an active ligament regeneration process similar to that of the autograft.

Solid healing between the tendon and bone tunnel is essential to successful ACL reconstruction [50]. The mechanical properties of the femur-graft-tibia complex are determined by the mode of fixation in the bone tunnels, especially shortly after the operation [51]. In our study, screws located about 5 mm from the bone tunnels were used to fix the two ends of graft in the bone. This anchoring technique facilitated immediate joint stability after surgery, which is essential for an animal model without leg restraint. Additionally, this anchoring technique does not disturb the physiological healing process between bone and the graft. Shinya Oka *et al*. reported that bone ingrowth into the tendon-bone interface determined the size of the bone tunnel area [52]. Bone tunnel area was evaluated using micro-CT to assess tendon-bone healing in our study. Regenerated ligament tissue filled the bone tunnel, and no mineralized tissue was detected in the bone tunnel 4 weeks postoperatively. Micro-CT evaluation showed the bone tunnel area of the autograft group to be larger than that of the scaffold group. The silk-collagen scaffold was a poriferous biomaterial capable of inducing easy cell infiltration. Histology staining revealed the formation of trabecular bone at the tendon-bone interface at 16 weeks postoperatively. Subtle changes were discerned with micro-CT scan, and gross information on the newly formed mineralized tissue in bone tunnels was collected through the micro-CT images. There were two types of tendon-bone healing, one showing that graft and bone came into direct contact with each other through Sharpey’s fibrous insertion. The other type showed normal tendon-bone insertion with layered chondral formation at the graft-bone interface [53]. Typical Sharpey’s fibers were observed in the autograft group 16 weeks after surgery, which was consistent with the scar healing process. In the scaffold group, mature trabecular bone grew into the inside of the graft, demonstrating that the silk-collagen graft could induce bone ingrowth. The results were consistent with those from micro-CT and biomechanical assessment.

Many studies have demonstrated that silk is degradable via proteolytic degradation mediated by a foreign body response. The degradation process occurs over a long period and leads to a decrease in the tensile strength of the scaffold. The reported loss of tensile strength has ranged from 55% at 40 days to 73% at 30 days after the scaffolds were implanted subcutaneously [25, 54]. The loss rate of tensile strength is mainly determined by the structures of scaffold, the implantation site, the mechanical environment, and the physiological status of the host [48]. The synovial environment of the articular cavity is very different from the subcutaneous environment because of its low vascularity. Failure load and stiffness are two important parameters for a ligament produced through tissue engineering. Because of the degradation of the scaffold and the infiltration of newly formed tissue, the failure load and stiffness at 16 weeks postoperatively mainly reflected the mechanical properties of the regenerated fibrous tissue and scaffolds. The cells on the scaffold produced ECM, including proteoglycans and collagen fibers, which compensated for the loss of tensile strength caused by degradation.

## Conclusion

In the present study, a scaffold incorporating collagen sponges into knitted silk fibroin mesh was fabricated to mimic ligament components. The findings of this study suggest that the silk-collagen scaffold was suitable for ACL reconstruction in our rabbit model, and it has great possibilities for future clinical application.
